# National Data Analysis and Systematic Review for Human Resources for Cervical Cancer Screening in Japan

**DOI:** 10.31557/APJCP.2021.22.6.1695

**Published:** 2021-06

**Authors:** Chisato Hamashima, Seiju Sasaki, Satoyo Hosono, Keika Hoshi, Takafumi Katayama, Teruhiko Terasawa

**Affiliations:** 1 *Health Policy Section, Department of Nursing, Faculty of Medical Technology, Teikyo University, 2-11-1 Kaga, Itabashi-ku, Tokyo 173-1211, Japan. *; 2 *Center for Preventive Medicine, St. Luke’s International Hospital, 8-1 Akashi-cho Chuo-ku, Tokyo 104-6591, Japan. *; 3 *Cancer Screening Assessment Section, Division of Screening Assessment and Management, Center for Public Health Science, National Cancer Center, 5-1-1 Tsukiji Cyuo-ku, Tokyo, 104-0045, Japan.*; 4 *Center for Public Health Informatics, National Institute of Public Health, 2-3-6 Minami, Wako 351-0197, Japan. *; 5 *Department of Hygiene, Kitazato University School of Medicine, 1-15-1 Kitazato Minami-ku, Sagamihara, Kanagawa, 252-0374 Japan. *; 6 *College of Nursing Art and Science, University of Hyogo Prefecture, 13-71 Kita-Ohji, Akashi 673-8588, Japan. *; 7 *Section of General Internal Medicine, Department of Emergency and General Internal, Medicine, Fujita Medical University School of Medicine, Toyoake, Aichi 470-1192, Japan. *

**Keywords:** Cervical cancer, cancer screening, human resources, gynecologist, pap smear, smear taker

## Abstract

**Background:**

Although cervical cancer screening has been performed as a national program since 1983 in Japan, the participation rate has remained below 20%. Equity of access is a basic requirement for cancer screening. However, taking smears from the cervix has been limited to gynecologists or obstetricians in Japan and it might be a barrier for accessibility. We examined the current access and its available human resources for cervical cancer screening in Japan.

**Methods:**

We analyzed the number of gynecologists and obstetricians among 47 prefectures based on a national survey. A systematic review was performed to clarify disparity and use of human resources in cervical cancer screening, diagnosis, and treatment for cervical cancers in Japan. Candidate literature was searched using Ovid-MEDLINE and Ichushi-Web until the end of January 2020. Then, a systematic review regarding accessibility to cervical cancer screening was performed. The results of the selected articles were summarized in the tables.

**Results:**

Although the total number of all physicians in Japan increased from 1996 to 2016, the proportion of gynecologists and obstetricians has remained at approximately 5% over the last 2 decades. 43.6% of municipalities have no gynecologists and obstetricians in 2016. Through a systematic review, 4 English articles and 1 Japanese article were selected. From these 5 articles, the association between human resources and participation rates in cervical cancer screening was examined in 2 articles.

**Conclusions:**

The human resources for taking smears for cervical cancer screening has remained insufficient with a huge disparity among municipalities in Japan. To improve accessibility for cervical cancer screening another option which may be considered could be involving general physicians as potential smear takers.

## Introduction

Cervical cancer screening has commonly performed worldwide because of the heavy burden of the disease. Although the mortality from cervical cancer has decreased and has subsequently flattened in Japan, the incidence of cervical cancer has slightly increased over the last decade (National Cancer Center, 2020). In 2017, the age-standardized incidence by world population was 11.7 (/100,000) and age-standardized mortality was 2.2 (/100,000). Similar trends of mortality from cervical cancer have been observed in developed countries (IARC, 2020). Incidentally, most of these developed countries have established national programs for cervical cancer screening and have maintained high participation rates (Elfström et al., 2015). 

Although cervical cancer screening has been performed as a national program in Japan since 1983 (Hamashima, 2018), the participation rate has remained lower than those of other developed countries (OECD and European Union, 2018). The national average participation rate of cervical cancer screening in communities has gradually increased, although it has remained below 20% in population-based screening (Ministry of Health, Labour and Welfare, Tokyo, 2017). Accessibility has not been considered even if it is one of the most important factors in keeping equity for cancer screening (IARC, 2019; Andermann et al., 2008). Facilities for diagnosis and treatment should be equally available to achieve the goal of cervical cancer screening. Although there is a continuous discussion to improve participation rate, discussions regarding the needed resources for cancer screening programs have remained scared in Japan. In cervical cancer screening, taking smears and precancer treatments are basic processes and these roles mainly depend on gynecologists. In rural areas in Japan, the insufficient number of clinicians, particularly gynecologists and obstetricians, has become a social problem (Nakagi et al., 2010). We focused on human resources for cervical cancer screening and the present situation was analyzed through the national survey and previous studies in Japan. 

## Materials and Methods

To clarify the burden of accessibility to cervical cancer screening, we examined and analyzed disparity of the number of gynecologists and obstetricians among prefectures in Japan. A systematic review was performed to examine disparity and use of human resources in cervical cancer screening, diagnosis, and treatment for cervical cancers. 

*National data analysis of numbers of gynecologists as human resources*

The Japanese government conducts a national survey every 2 years to determine the number of physicians, dentists, and pharmacists who work in Japan as well as their actual working conditions (Ministry of Health, Labour and Welfare, 2020-a). The information obtained for physicians includes their main specialty, medical working facilities, and their working locations. The current status of human resources for cervical cancer screening was also clarified by determining the number of gynecologists and obstetricians from the above-mentioned national survey. Obstetricians were included as human resources for cervical cancer screening because their roles have been nearly equal to those of gynecologists in local areas. First, the trend of the number of all clinicians, gynecologists, and obstetricians per 100,000 population was observed from 2000 to 2016. Second, the number of gynecologists and obstetricians per 100,000 women in 2016 was recalculated using the resident registered population in 2016 as denominators (Ministry of Health, Labour and Welfare, 2020-b). The denominator was limited to women aged 20-69 years which was an actual target group for cervical cancer screening. The results were compared among the 47 prefectures. The numbers of municipalities without gynecologists and obstetricians were also calculated.

Theses information did not include personal information. Based on the ethical guidelines for medical and health research involving human subjects developed by the Japanese government, in the present study, secondary data from the national database were used, thus informed consent was waived.

*Systematic review*

To identify the involved human resources and their impact on cervical cancer screening, in Japan, we selected articles that included any of the following issues and grouped them into 3 categories: 1) association between municipalities with and without gynecologists and participation rates in cervical cancer screening, 2) distribution of special hospitals with certified gynecological oncologists, and 3) comparison of treatment results (survival rate) with and without certified gynecologists. Although the first category is associated with the participation rate for cervical cancer screening, the second and third factors are not directly associated. However, these factors lead to regional disparity if they are insufficient.

We searched articles that may fall under the above-mentioned 3 categories using Ovid-MEDLINE and Ichushi-Web (Igaku Chuo Zasshi). The search terms mainly used were ‘uterine cervical neoplasms’, ‘uterine cervical dysplasia’, ‘gynecologist’, ‘oncologist’, ‘specialty’, ‘physician’, ‘family practice’ or ‘Japan’ until the end of January 2020. The detailed information for making the searches on Ovid-MEDLINE and Ichushi-Web is described in the Supplementary file. The languages of the article included were only English and Japanese because the main topic was limited to the Japanese context. Original articles published after peer-review were included, whereas guidelines, evidence reports, conference proceedings, and abstracts were excluded. 

To select the appropriate evidence for our research questions, we performed a two-stage review: the title and abstract were initially checked, and the selected full-text articles were subsequently reviewed. For the initial step, articles without an abstract were excluded. Two reviewers screened the abstracts and titles individually and subsequently reviewed the full texts of potentially relevant studies. If the decision for the text review was inconsistent, the appropriateness of these studies was carefully discussed during meetings. The process for a systematic review was confirmed by the PRISMA 2009 Checklist. Finally, the articles which reported accessibility in primary screening were selected from 3 categories which assessed human resources in gynecological clinical practices. 

## Results

*Human resources for cervical cancer screening*

**Figure 1:**
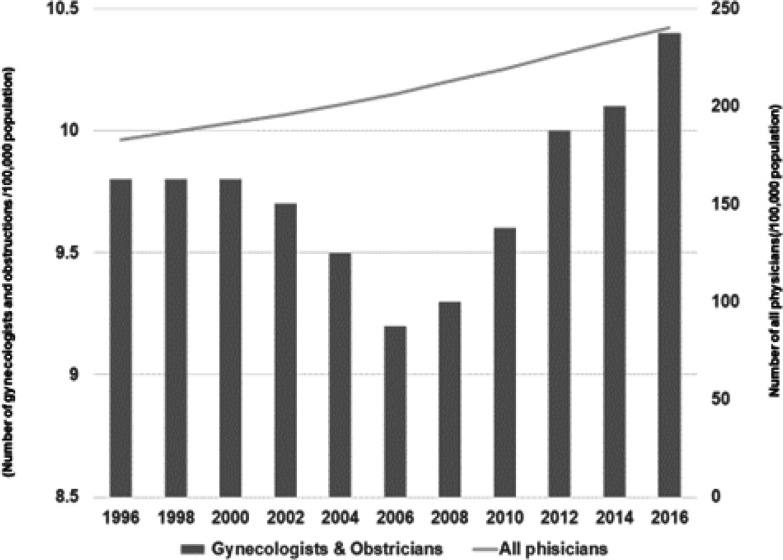
Trends of the Number of Physicians. From 1996 to 2016, the total number of all physicians were increased. However, for two decades, the proportion of gynecologists and obstetricians has remained at approximately 5%.

**Figure 2:**
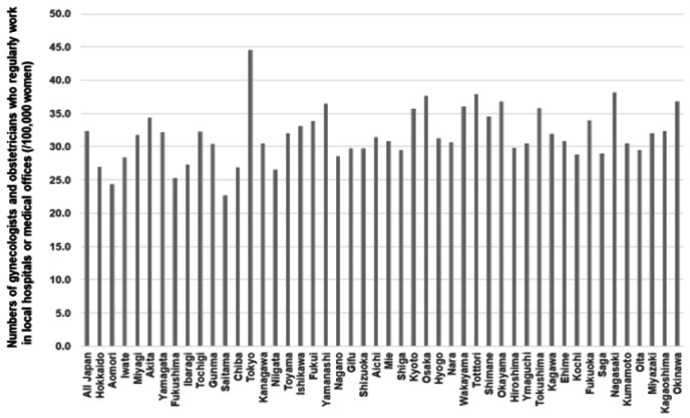
Comparison of the Number of Obstetricians and Gynecologists among 47 Prefectures. The national average of the number of gynecologists and obstetricians was 32.3 (/100,000 women aged 20-69 years). In most prefectures, the number was below the national average. The numbers were cited from the Survey of Physicians, Dentists and Pharmacists 2016

**Figure 3:**
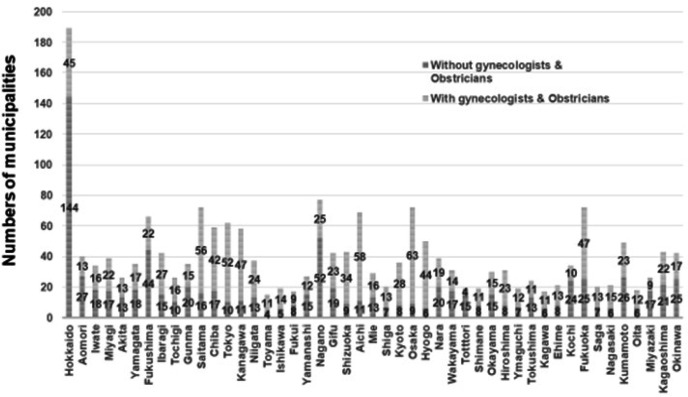
Numbers of Municipalities without Gynecologists and Obstetricians who Regularly Work in Local Hospitals or Medical Offices. There were 43.6% of municipalities without gynecologists’ obstetricians who regularly work in local hospitals or medical offices. The numbers were cited from the Survey of Physicians, Dentists and Pharmacists 2016

From 1996 to 2016, the total number of all physicians increased; however, for two decades, the proportion of gynecologists and obstetricians has remained at approximately 5% (Figure 1) (Ministry of Health, Labour and Welfare, 2020-a). Although the numbers of gynecologists and obstetricians decreased until 2006, these numbers have recovered recently. In 2016, the total numbers of all physicians were 240.1 (/100,000 population) and 10.4 (/100,000 population) for gynecologists and obstetricians, respectively (Ministry of Health, Labour and Welfare, 2020-a). However, only 16 prefectures exceeded the national average of gynecologists and obstetricians in 2016 (Figure 2). The numbers of gynecologists and obstetricians were higher in western Japan than in eastern Japan. Even if the prefectural average exceeded the national average, there were still municipalities that had no gynecologists and obstetricians (Figure 3). There were 43.6% municipalities with gynecologists and obstetricians who did not work in local medical facilities (Ministry of Health, Labour and Welfare, 2020-a). Although Tokyo had the highest number of gynecologists and obstetricians among the prefectures, 10 out of 62 municipalities were without gynecologists and obstetricians. In Hokkaido, the proportion of municipalities without gynecologists and obstetricians was 76.2%. 

*Literature Search*

**Figure 4:**
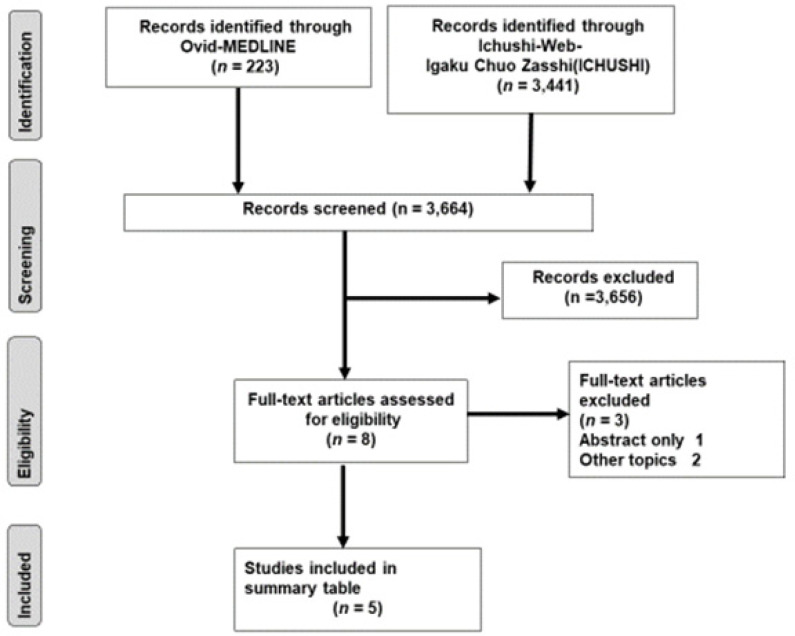
The Selection Process of Articles in the Literature Search Using the PRISMA 2009 Flow Diagram. The number of articles identified from the literature search using Ovid-MEDLINE and Ichushi-Web was 3,664 articles after a two-stage review, 4 English articles and 1 Japanese article were selected

The total number of articles identified from the literature search using Ovid- MEDLINE and Ichushi-Web was 3,664 articles (Fig. 4). After a two-stage review, 4 English articles and 1 Japanese article were selected. From these 5 articles, the association between human resources and participation rates in cervical cancer screening was examined in 2 articles (Table 1). One article reported on the national distribution of gynecologists (Table 2). Two articles analyzed the treatment results between the hospitals with and without certified gynecologists.

*Association of human resources with participation rates in cervical cancer screening*

One article analyzed the association of human resources with participation rates among the municipalities in Fukushima prefecture, and another study used the national data (Table 1). Morimura et al. compared the participation rates in 2 types of municipalities: those that allowed only cervical cancer screening in their own municipalities and those that permitted collaboration with neighboring municipalities that had medical facilities with gynecologists. In the latter municipalities, the opportunities for cervical cancer screening were increased (Morimura et al., 2007). Moreover, the participation rate was significantly higher in the latter municipalities than in the former municipalities (6.5% vs 3.9%, p < 0.01). In addition, when the periods for cervical cancer screening extended, a significant increase in the participation rate was observed (correlation = 0.322, p < 0.01). Sano et al. reported the association of the participation rate with the number of gynecologists (Sano et al., 2017). Marginal effects were observed in that the participation rate was significantly increased by only 2.54 percent points in all municipalities when 1 gynecologist per 1,000 women was available. These marginal effects were emphasized in rural municipalities, and an increase of 3.68 percent points was expected under the same condition.

*Treatment results and certified gynecological oncologists*

To improve treatment results and provide high-quality diagnosis and treatment, the Japan Society of Gynecologic Oncology (JSGO) has developed programs and accredited gynecological oncologist experts mainly based on their operative experiences (Mikami et al., 2018). JSGO has also accredited hospitals in which certified gynecological oncologists regularly work (Fujii, 2016).

Two articles reported the difference of survival rates with and without gynecological oncologists (Table 2). Mikami et al. reported the treatment results of accredited hospitals (Mikami et al., 2018). The survival rates of cervical cancer patients 2,500 days after their initial treatments were significantly higher in the JSGO-accredited hospitals than in the JSGO-nonaccredited hospitals (73.3% vs 68.7%, p < 0.01). Yagi et al. also reported similar results of the 5-year survival rates among hospitals that had a different number of gynecological oncologists (Yagi et al., 2019).

Despite the nationwide distribution of gynecologic oncologists in Japan, their number has remained insufficient in most prefectures (Fujii, 2016). If the gynecologists were restricted to certified gynecologists, there was a huge disparity in accessing high-quality treatment for cervical cancer. There were only 4 leading prefectures with 1.4 to 2.0 certified gynecologists per 100,000 women, namely, Toyama, Nara, Tottori, and Fukuoka. Most prefectures had less than 0.7 certified gynecologists per 100,000 women. This insufficient number of gynecologists was shown in the national survey by academic society, which became an obstacle to verifying the effects of cervical cancer screening.

**Table 1:** Association of the Number with Gynecologists and Participation Rate in Cervical Cancer Screening

Authors	Year published	Target region	Number of municipalities	Number of gynecologists	Participation rate	Main results	Association of number of gynecologists with participation rate in cervical cancer screening
Morimura Y, et al. (12)	2007	Fukushima Prefecture	64	Unclear	Collaboration municipalities (36) 6.49% Non-Collaboration municipalities (28) 3.92%	The participation rate of municipalities collaborating with neighboring municipalities was significantly higher than that of municipalities not collaborating with neighboring municipalities (p < 0.01).	Positive
Sano H, et al. (13)	2017	All Japan	1469	0.151(/1000 women)	60.60%	The marginal effect of the number of gynecologists per 1,000 women was significantly positive in all municipalities (2.54 percent points) and rural municipalities (3.68 percent points).	Positive

**Table 2:** Summary of the Studies Related to Certified Gynecologists and Hospitals

Authors	Year published	Target region	Certification	Certification number (year)	Main outcomes	Main results
Fujii T (14)	2016	All Japan	Gynecological oncologists	720 (2012)	Regional distribution certified gynecologists	There was a huge disparity in the medical facilities with certified gynecologists who regularly worked in the facilities.
Mikami M, et al. (15)	2018	All Japan	Hospitals	119 (2006)	Survival rate (2500 days)	The survival rates of cervical cancer patients in 2500 days after their initial treatments were significantly higher in the JSGO-accredited hospitals than non -accredited hospitals (73.3% vs 68.7%, p < 0.01).
Yagi A, et al. (16)	2019	All Japan	Hospitals	147(2010)	5-year survival rate	The 5-year survival rates were higher in hospitals with 2-or more gynecological oncologists than those with 0 or 1 gynecological oncologists (79.0% vs 75.4%, p<0.01).

JSGO, Japan Society of Gynecologic Oncology

## Discussion

Based on the national survey, a huge disparity was recognized in the human resources of cervical cancer screening. Sano et al., (2017) reported a significant correlation between the number of gynecologists and the participation rate in cervical cancer screening at municipal level. To compensate for the insufficient number of gynecologists, collaboration with neighbor municipalities and the use of mobile clinics have supported to provide an opportunity for cervical cancer screening. The disparity of human resources is also affected by the survival rate of cervical cancer (Mikami et al., 2018; Yagi et al., 2019). Although gynecologists must cover from screening to treatment for cervical cancer, the numbers have been definitively insufficient to match the suited to increase of participants in cervical cancer screening. 

Accessibility is defined as one of the basic requirements to maintain equity for the target population in cancer screening programs (Andermann et al., 2008). However, in Japan, there has been insufficient discussion regarding the resources for cancer screening programs particularly with the introduction of new techniques (Hamashima and Goto, 2017). In the U.S. and European countries, the capacity of colonoscopy has been investigated since the introduction of colorectal cancer screening using fecal occult blood testing and total colonoscopy (Seeff et al., 2004-a and 2004-b; Lau and Gregor, 2007; van Turenhout et al., 2012; Joseph et al., 2016; Comas et al., 2016). Since the announcement of the cancer control plan to increase the participation rate in cancer screening programs by the Japanese government (Ministry of Health, Labour and Welfare, 2017), various promotion strategies including sending invitation letters and conducting awareness campaigns have been attempted (Sano et al., 2014; Hirai et al., 2016). Although improvement of participation rate in cancer screening has been huge concern, only 2 articles investigated the association between participation rate and human resources in cervical cancer screening regardless of their long history since the introduction. 

Gynecologists are expected to take the primary role in making a diagnosis and planning the treatment during cervical cancer because of their special knowledge and techniques. Though the systematic review, three articles were found, which evaluated distribution of special hospitals with certified gynecological oncologists, and comparison of survival rates with and without certified gynecologists (Mikami et al., 2018; Yagi et al., 2019). Despite the nationwide distribution of gynecologic oncologists in Japan, their number has remained insufficient in most prefectures (Fujii, 2016). Thus, their number has remained insufficient to fill each critical role. Cervical cancer screening methods are usually simple, and clinicians can perform them regardless of their expertise. Taking smears has been traditionally limited to gynecologists and obstetricians since the introduction of cervical cancer screening in Japan. In actual, mobile clinics have been very useful in compensating for the insufficient opportunities for cervical cancer screening. Besides, the number of certified gynecological oncologists has also remained insufficient, and their distribution has been biased (Sano et al., 2017). As a result, women with abnormal smear have not easily access to gynecologists. However, based on the recent trend, a rapid increase in the number of gynecologists cannot be expected. 

In order to keep equal access, one possible solution may be for gynecologists to share screening work with general physicians in regional areas. In several countries, medical systems that permit general physicians and midwives to take smears for cervical cancer screening are now seeing the benefit (Yabroff et al., 2009; McDonald et al., 2001; Ideström et al., 2007; Cooper and Saraiya, 2014; Poncet et al., 2016). These systems can help improve access to cancer screening programs, although they can also serve as a barrier to referring abnormal results to gynecologists. In Sweden, midwives have the responsibility of taking smears during cervical cancer screening and of simultaneously conducting consultations on health problems (Ideström et al., 2007). The European guidelines which established the basic concept of quality assurance referred to organized screening systems including smear taking (Arbyn et al., 2007). Following this, the guidelines for quality assurance have been published in the United Kingdom and Australia, and these guidelines also included the basic requirement of smear taking (Cancer Council Australia, 2016; Public Health England, 2016). In England, the NHS Cervical Screening Programs (NHSCSP) has also provided education and training programs for smear takers including general physicians, nurses and midwives (Public Health England, 2016). Candidate for smear taking have lectures and practical training for their techniques. Providing opportunities for training and management guidelines could aid in that appropriate smear are taken for cervical cancer screening. Then, scare number of gynecologists can then intensively focus on colposcopic examination, treatment and surveillance after treatment of precancerous lesions. From the perspective of resource allocation, sharing of the roles between specialist and general physician can improve women’s access to screening, diagnosis and treatment.

Recent studies have reported that self-sampling HPV testing is a useful approach to increasing the participation rate (Arbyn et al., 2018). Also, the sensitivity and specificity of self-sampling HPV testing are nearly equal to those of a clinician performing the HPV testing (Arbyn et al., 2018; Polman et al., 2019; Gustavsson et al., 2019). Some countries have already introduced self-sampling HPV testing for non-attenders (Health Council of the Netherlands, 2016; Medical Services Advisory Committee, 2013; Danish Health and Medicines Authority, 2017). It is also used in low-resource areas with poor access to screening services (Zhao et al., 2012). The introduction of self-sampling HPV testing can be a viable option for reducing the workload of gynecologists in taking smears. Besides, self-sampling HPV testing can also be adopted in rural areas without gynecologists and obstetricians.

There are some limitations to this study. First, the analysis of human resources was a descriptive study based on the national survey. Mutual collaboration of neighboring municipalities with and without gynecologists in local areas were shown except Fukushima study. To clarify the appropriate supply of cervical cancer screening, the demands and collaboration in local areas should be investigated at the municipality level. Second, we could find only 2 articles which assessed human resources for cervical cancer screening. This topic might be discussed in conference proceedings or some reports for cancer screening in local level. Since publication criteria was limited to original article written by English or Japanese, the information was limited. However, in other screening programs, there is few discussions of the lacking available human resources in Japan. The role of general physicians in cancer screening programs has become important because they also take charge of general health check-up collaborated with cancer screening in local areas. Finally, the topic issue is limited to the local problem in Japan. However, our experience is informative to share a similar problem of cervical cancer screening in Asian countries.

To date, there has been a huge deficiently and disparity in human resources for taking smears for cervical cancer screening in Japan. In the series of medical procedures from screening to diagnosis and treatment, division of roles and collaboration with general physicians should be considered for the efficient use of limited resources for cervical cancer screening. To improve disparity in the accessibility to cervical cancer screening, a further study including capacity estimation based on the local demand is warranted.

## Author Contribution Statement

CH designed the study. KH performed literature searches and summarized the search terms. CH, SS, KH, SH, TK, and TT performed a systematic review. CH conducted a statistical analysis of the data. CH wrote and made critical revisions to the article. All authors read and approved the final manuscript.
